# Actual Causes of Death in Relation to Media, Policy, and Funding Attention: Examining Public Health Priorities

**DOI:** 10.3389/fpubh.2020.00279

**Published:** 2020-07-07

**Authors:** Meagan R. Pilar, Amy A. Eyler, Sarah Moreland-Russell, Ross C. Brownson

**Affiliations:** ^1^Prevention Research Center in St. Louis, Brown School at Washington University in St. Louis, St. Louis, MI, United States; ^2^Department of Surgery, Division of Public Health Sciences and Alvin J. Siteman Cancer Center, Washington University School of Medicine, Washington University in St. Louis, St. Louis, MI, United States

**Keywords:** actual causes of death, prioritization, resource allocation, media, policy, funding

## Abstract

Despite numerous public health advancements over the last century, we continue to under-invest in prevention and public health efforts. As a result, one of the most challenging aspects of public health is prioritizing the use of limited resources. Building on the foundation of previous researchers, the goal of this exploratory study was to provide current estimates for the actual causes of death, media attention, policy focus, and research funding in the United States. In addition, we sought to calculate and compare media attention, policy attention, and research funding trends to better assess the nation's prioritization of health issues. Using a systematic approach, we searched available databases, including Media Cloud, Nexis Uni, Congress.gov, and the Department of Health and Human Services Tracking Accountability in Government Grants System from January 1, 2010-December 31, 2019 and compared how the actual causes of death in the United States align with health-related media attention, policy attention, and federal spending. Overall, our findings suggest that our priorities are not well-aligned with the actual causes of death. Certain actual causes appear to be consistently misaligned across media, legislative, and financial sectors (e.g., tobacco). This work highlights the importance of multiple strategies—media coverage, national legislation, and government spending—as indicators of public health attention and priorities. These results may inform discussions about how to best allocate U.S. public health resources to better align with the actual causes of death.

## Introduction

Over the last century, public health has contributed to drastic reductions in mortality and morbidity. In spite of these advancements, we continue to under-invest in prevention and public health efforts (1). In 2018, the United States spent $3.6 trillion dollars on healthcare, ~18% of the country's gross domestic product (2). However, <3% of total healthcare spending in the United States is dedicated to public health and prevention each year (3–5). In 2018, this equated to an average of $10,874 per person in treatment costs, compared to only $286 per person in prevention efforts (6).

Underinvestment in prevention and limited resources often require decision makers to place priority on some efforts over others. There are several factors influencing prioritization. One way to determine priority is the simple quantification of the problem. When quantifying and prioritizing public health issues, there are many different scientific measures, including counting total deaths, preventable deaths, DALYs (disability-adjusted life years) and QALYs (quality-adjusted life years) (7, 8). Another factor that may shift priorities for resource allocation is media attention, which can influence awareness of certain health issues (9, 10). This, in turn, can shape the public's perception of current health issues (11). Policy legislation—both proposed and enacted—is another measure of the extent of public health action. Finally, government funding allocated for prevention and public health is an indicator of the relative priority of an issue.

Each of these indicators—media coverage, national policies, and government spending—are intertwined within the policy process. Howlett and Ramesh outline the policy process in the following steps: defining the problem; agenda setting; policy formulation; policy implementation; and policy evaluation (12). Though many scholars acknowledge the oversimplification of the policy process within this model (13), it can be helpful in this case when considering how media, national policies, and government spending fit within the complex policy process. Media attention can greatly impact early stages in the policy process, such as agenda setting (14–17), as well as policy formulation, evaluation, and termination (18). Policy development and implementation consequently impact government spending for health-related issues (19). As a result, media coverage, national legislation, and government spending may be related—but distinct—indicators of the nation's public health priorities.

Using recent infectious diseases as an example, we can examine how prioritization in media, policy, and research spending can interact to influence a public health issue. The novel Coronavirus disease (COVID-19), a community-spread respiratory illness, was first detected in China but has since spread to more than 100 countries worldwide, reaching pandemic status and receiving substantial media attention in the United States (20–23). By May 1, 2020, after the virus had exceeded containment measures, more than 70 new pieces of legislation were brought before Congress, one of which was passed (24). This piece of legislation provided $8.3 billion in funding for COVID-19 control and research through federal agencies. Given the large-scale impact of COVID-19 in the United States (25), multiple strategies (media, legislation, and government funding) have been used to target a public health crisis. Though the vast impacts of this pandemic are ongoing, COVID-19 provides an example of quickly mobilizing national resources to impact an emerging infectious disease.

A combination of strategies, such as media, policy, and funding, can also affect chronic disease prevention and control efforts [e.g., obesity, tobacco, cancer early detection; (26–28)]. For example, beginning in 1989, California's tobacco control initiative funded two primary strategies to decrease smoking rates—media and policy advocacy (29). The efforts included mass media campaigns, statewide cigarette taxes, and increased funding for prevention and treatment programs. The combined approach resulted in decreased smoking prevalence (29).

In 1993, McGinnis and Foege described the burden associated with the leading causes of death in the United States and analyzed the underlying modifiable risk factors (30). They named these factors the “actual causes of death” and estimated the annual total number of deaths due to each actual cause. One of the primary lessons from this study was the need for a greater emphasis on prevention efforts, as well as a redistribution of public health resources in the United States (30). Mokdad et al. updated the data in 2004 and found that the leading actual causes of death for U.S. residents were tobacco (435,000 deaths/year), diet/physical activity (400,000 deaths/year), and alcohol consumption (85,000 deaths/year) (31). From these estimates, we see that the top four actual causes of death are responsible for nearly 40% of all annual deaths in this country. Given that many of the actual causes of death are modifiable risk behaviors with underlying environmental and policy determinants (32), this presents an excellent opportunity for examining health priorities and potential interventions. However, there has, to our knowledge, been no further exploration of coverage of health topics and policy actions compare with the actual causes of death.

Increased media attention, policy mandates, and government-funded research contribute to the control and containment of large-scale public health concerns, but the prioritization of resources may differ from actual causes of death. For example, there may be variations in resource allocation based on the amount of media attention between emerging infectious diseases and chronic diseases. Since resource allocation decisions are complex, we need to explore how other contributing factors, such as media attention, legislation, and spending, align with the actual causes of death.

Building on previous research (30, 31), the goals of this study were two-fold. First, we used a systematic approach to provide current estimates for the actual causes of death, media attention, policy attention, and research funding in the United States. Next, we described how the actual causes of death in the United States align with health-related media attention, policy attention, and federal spending.

## Materials and Methods

### Search Strategy

The search terms included in this review were informed by the nine categories described by McGinnis and Foege—tobacco; poor diet and physical inactivity; alcohol consumption; microbial agents; toxic agents; motor vehicles; firearms; sexual behavior (e.g., sexually transmitted diseases and HIV); and illicit drug use (30). However, for the purposes of this paper, we separated *poor diet* and *physical inactivity* to analyze the risk factors independently, since they are independent risk factors for multiple chronic diseases (33). Beyond that change, we used the original categories provided by McGinnis and Foege for this analysis; updating the original categories was beyond the scope of this work. Then MeSH terms with similar meanings were added to ensure a more inclusive search; see Supplementary Table 1 for a complete list of terms. These search terms were applied to the following databases: the Institute for Health Metrics and Evaluation (IHME) (34), Media Cloud (35), Nexis Uni (36), Congress.gov (24), and the Department of Health and Human Services (HHS) Tracking Accountability in Government Grants System (TAGGS) (37). All database searches were restricted by geography, language, and time frame; that is, only results that were published in the United States (i.e., 50 states and Washington, D.C.), written in English, and distributed from January 1, 2010–December 31, 2019 were analyzed. More detail regarding search strategies is provided in Supplementary Table 2.

### Actual Causes of Death

The IHME, a website which provides population-level health data as part of research being conducted at the University of Washington, was used to update total deaths due to the actual causes of death (34). The Global Burden of Disease Compare feature provided 2017 data regarding deaths attributable to each actual cause of death in the United States. Individual searches were completed for each cause of death using the previously described search terms. The IHME database houses information for all deaths and classifies them as either direct *causes of death* or *risk factors*, which refers to potentially modifiable factors (34). The majority of the actual causes of death were classified by the IHME as *risk factors* with the exception of *microbes, motor vehicles*, and *firearms*, which were classified as *causes of death*. This difference in classification did not affect the search strategy but may be useful if trying to replicate study findings.

### Media

Media Cloud and Nexis Uni were selected as databases for media analysis. Media Cloud was chosen because it specializes in tracking online media presence, while Nexis Uni provides data on a variety of media, including national- and local-level newspapers. These two databases were chosen to create a more comprehensive, complementary approach to media analysis. The Media Cloud database encompasses online news stories, as well as hyperlinks, Bitly clicks, and social media shares from more than 60,000 online sources (35). Using the Explorer function of Media Cloud, we included both U.S. national and state/local parameters into our search and tracked the total number of daily occurrences for each cause of death from 2010 to 2019 using the previously described search terms. We calculated the total number of search results per year and created an overall average for each cause of death. Nexis Uni results, derived from more than 15,000 sources (36), were filtered by publication type to include only newspapers and newswires/press releases. We recorded the total number of references to each actual cause of death each year and averaged the totals. Finally, we used a Spearman rank correlation, a non-parametric measure of the strength and direction of an association between two variables, to examine the relationship between the number of deaths per year and the amount of media attention in both Media Cloud and Nexis Uni databases. Each result from these databases was given equal weight during analysis, as the goal was to analyze the overall quantity of media attention.

### Policy

Congress.gov was selected as a source of data because it allowed us to restrict our search to only legislation [i.e., no congressional records, treaty documents; (24)]. We then compared the statuses (i.e., legislation introduced vs. legislation that became law) and rates of legislation across the actual causes of death. Total numbers of proposed and passed legislation were calculated for January 1, 2010–December 31, 2019, and an overall average was calculated. Finally, we ranked the causes of death by number of proposed and passed legislation and conducted a Spearman rank correlation to assess the alignment with the number of deaths per year. Because our goal was to analyze overall quantity of proposed and passed legislation, each result from this database was given equal weight during analysis.

### Federal Funding

TAGGS, a website that compiles financial assistance (i.e., grants and contracts) provided by the HHS Operating Divisions and the Office of the Secretary Staff Divisions (37), was selected as a way to gauge government spending on research and development. It is important to note that the search strategies for this particular database differed from the others, so the search terms were modified slightly. For example, truncated terms were not permitted in TAGGS. As a result, all truncated search terms were spelled out fully and listed (e.g., “pollute” and “pollution” instead of “pollut^*^”). Next, we were unable to narrow searches using Boolean operators, so terms that yielded irrelevant results were removed. For example, “shotgun” and “gun” search terms were removed from the *firearms* category because the terms yielded only funding related to aging (e.g., “whole genome shotgun sequencing”) and biotechnology (e.g., “electron gun”). Similarly, “cancer” was removed from the *sexual behaviors* category because it yielded results related to all forms of cancer—not just cancer related to sexual behavior (e.g., lung cancer). These terms would have skewed the appearance of funding opportunities related to firearms and sexual behaviors; all other search terms remained the same. The total amount of available funding per year was calculated for 2010–2019, and an overall average was calculated. Once again, we ranked the causes of death by amount of research funding allotted and conducted a Spearman rank correlation to assess the alignment with the number of deaths per year.

Finally, we calculated z-scores for each actual cause of death based on the ratio of number of totals deaths and the recorded media, policy, and federal funding presence using the following formula: Z = (x – μ)/σ. We first calculated a ratio between the total number of deaths (e.g., tobacco = 440,000) and the average amount of media, policy, or government spending for that cause of death. This ratio served as our raw score (x). Next, we subtracted the sample mean (μ) (i.e., the average of all ratios across all causes of death) from the raw score and divided by the population standard deviation (σ). These data provided a way to standardize scores across categories and directly compare the degree of misalignment between media, policy, and federal funding.

## Results

### Actual Causes of Death

First, we looked at the totals for causes of death, media attention, legislation, and government spending over time; the goal was to assess patterns and provide descriptive information. Table 1 presents a side-by-side comparison of the estimated totals for the actual causes of death collected by McGinnis and Foege (30) and Mokdad et al. (31), and the updated totals from IHME in 2017. As illustrated in Table 1, the total number of deaths from some causes, such as tobacco, has remained relatively stable from 1990 to 2017, while others, such as deaths from sexual behaviors, have decreased since 2000. Deaths related to toxic agents and illicit drugs have increased by more than 300 and 600%, respectively, since 2000. We then compared media coverage, legislation, and government spending with rankings of the 2017 actual causes of death in the United States.

**Table 1:** Estimated number of deaths for each actual cause of death in the United States 1990, 2000, 2017.

**Cause of death**	**No. (%) in 1990^**a**^**	**No. (%) in 2000^**b**^**	**No. (%) in 2017^**c**^**
Tobacco	400,000 (19)	435,000 (18)	440,000 (16)
Poor diet	300,000 (14)	400,000 (17)	503,000 (18)
Physical inactivity			73,000 (3)
Alcohol	100,000 (5)	85,000 (4)	81,000 (3)
Microbial agents	90,000 (4)	75,000 (3)	113,000 (4)
Toxic agents	60,000 (3)	55,000 (2)	201,000 (7)
Motor vehicles	25,000 (1)	43,000 (2)	30,000 (1)
Firearms	35,000 (2)	29,000 (1)	40,000 (1)
Sexual behavior	30,000 (1)	20,000 (1)	14,000 (1)
Illicit drug use	20,000 (<1)	17,000 (1)	105,000 (4)

a *Total of 2,148,463 deaths in the United States*.

b *Total of 2,403,351 deaths in the United States*.

c *Total of 2,813,503 deaths in the United States*.

### Media

**Figure 1:**
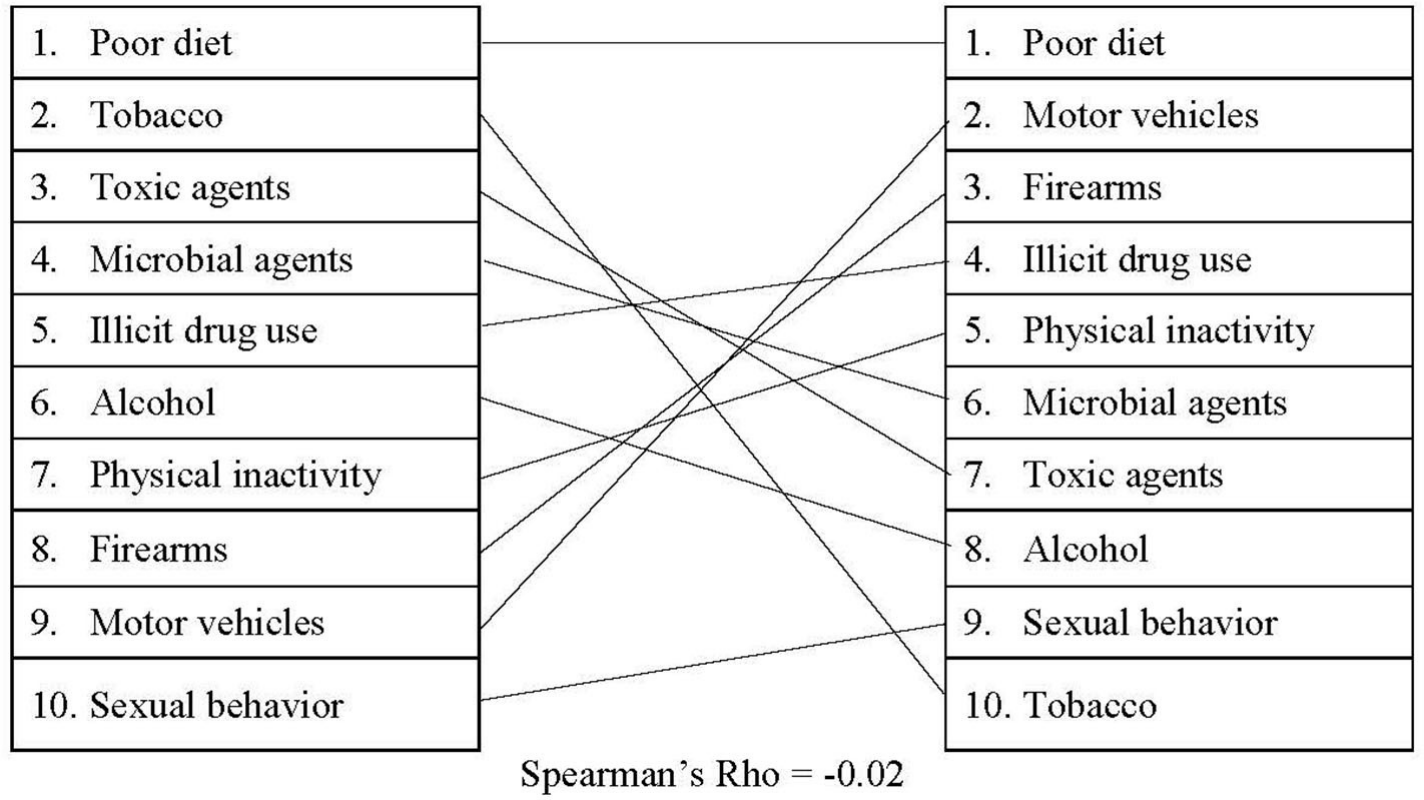
Comparing actual causes of death to Media Cloud presence.

The media search results between Media Cloud and Nexis Uni were not entirely aligned. Media Cloud, which collected media attention from a variety of sources at the national and local levels, suggested that media attention for the actual causes of death has increased steadily over time (see Supplementary Table 3 for detailed information). As displayed in Figure 1, when comparing the rankings between the actual causes of death and our Media Cloud search, there was a slight negative correlation (*r*_s_ = −0.02). In addition, poor diet and motor vehicles received the largest amount of media attention out of the actual causes of death on average (1,103,447 and 981,693 occurrences/year, respectively). However, motor vehicle deaths were relatively low in terms of mortality totals, accounting for <1% of all deaths in the United States. Tobacco received the lowest amount of attention on Media Cloud (168,706 occurrences/year), despite the fact that it was responsible for nearly 16% of all deaths in the United States in 2017. Similarly, our search criteria revealed that references to firearms appear in Media Cloud an average of 841,236 times/year. This is more media attention on average than tobacco, toxic agents, and microbial agents receive *combined*.

**Figure 2:**
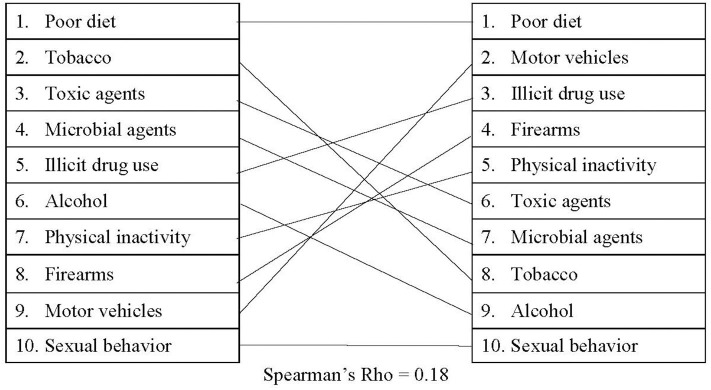
Comparing actual causes of death to Nexis Uni presence.

Our findings from the Nexis Uni database suggested that the cumulative media presence for the actual causes of death was relatively stable or has even decreased from 2010 to 2019 (see Supplementary Table 4 for detailed information), with a small, positive correlation (*r*_s_ = 0.18) between the actual causes of death and the Nexis Uni rankings. As shown in Figure 2, on average, sexual behavior, alcohol, and tobacco received the least media attention (41,541; 59,033; and 69,027 occurrences/year, respectively), while poor diet and motor vehicles received the most (283,053 and 205,656 occurrences/year, respectively). These results also show that the amount of media attention does not align with the total number of deaths per year. For instance, diet received more than four times as much media attention as tobacco did on average, despite the comparable death totals. In addition, illicit drug use generally received more than twice the media coverage that tobacco did, despite the fact that tobacco was responsible for nearly four times as many deaths.

### Policy

**Figure 3:**
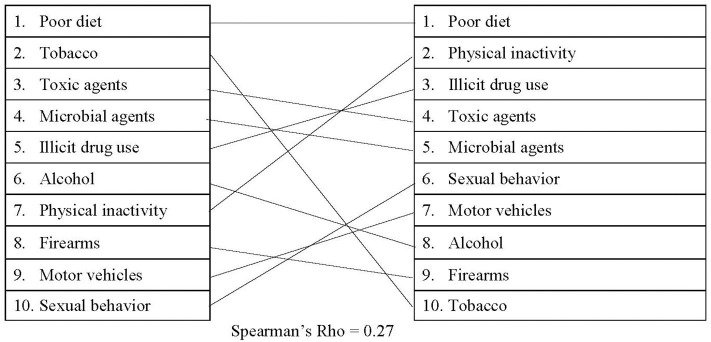
Comparing actual causes of death to proposed legislation.

The numbers of both proposed and passed legislation have remained relatively consistent from 2010 to 2019 for each actual cause of death (see Supplementary Tables 5, 6 for detailed information). As shown in Figure 3, results revealed a small, positive correlation (*r*_s_ = 0.27) between the actual causes of death and proposed legislation in the United States. The highest total number of proposed policies related to poor diet and physical inactivity, which elicited an average of 1,341 and 1,267 policy proposals/year, respectively. Alcohol, firearms, and tobacco, on the other hand, produced an average of 440, 428, and 398 policy proposals each year. Tobacco received only one-third of the total number of policy proposals compared to illicit drug use, despite a greater number of deaths being attributable to tobacco.

**Figure 4:**
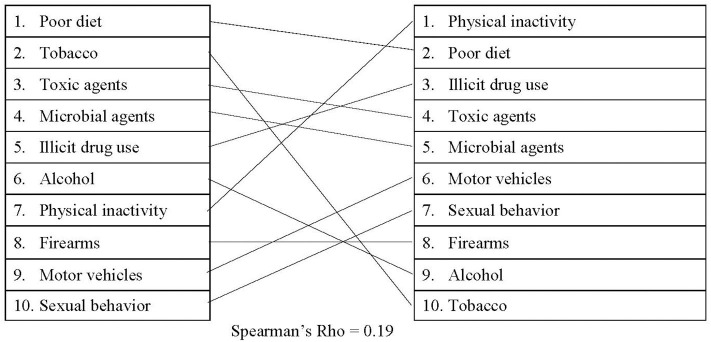
Comparing actual causes of death to passed legislation.

When comparing passed legislation with the actual causes of death, there was a small, positive correlation (*r*_s_ = 0.19), which is illustrated in Figure 4. On average, policies related to physical inactivity, poor diet, and illicit drug use had the highest approval totals (average of 50, 42, and 42 each year, respectively). Policies which had the lowest average number of approvals involved sexual behavior (20 policies/year), firearms (20 policies/year), alcohol (19 policies/year), and tobacco (17 policies/year). In 2017, physical inactivity and illicit drug use were responsible for a combined average of 178,000 deaths, which is significantly less than the deaths attributed to tobacco. However, more than double the number of policies were enacted each year for both physical activity and illicit drug use than for tobacco.

### Federal Funding

**Figure 5:**
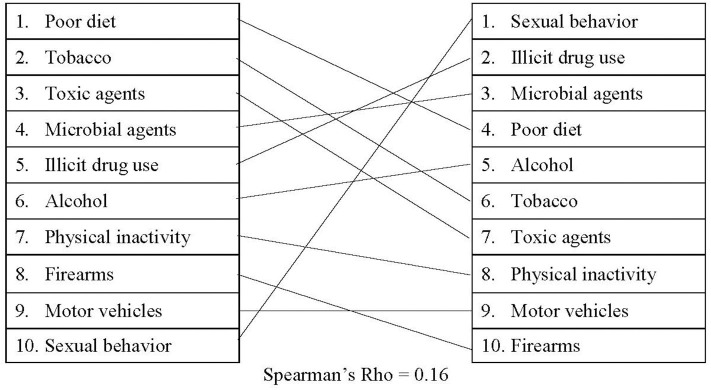
Comparing actual causes of death to government spending.

As shown in Figure 5, there was a small, positive correlation between the actual causes of death and federal funding (*r*_s_ = 0.16). For certain causes of death, the amount of federal funding dedicated to research and development has changed drastically over time (see Supplementary Table 7 for detailed information). For example, federal funding related to physical inactivity and sexual behaviors has remained relatively stable since 2010, while research related to illicit drug use has nearly doubled since 2010. Funding surrounding poor diet, on the other hand, has decreased ~33% between 2010 and 2019. An average of $5.4 billion was allotted to research surrounding sexual behaviors each year, making it the highest-funded actual cause of death. Similarly, an average of $4.4 billion in research funding was dedicated to illicit drug use, as well as $4.1 billion dedicated to microbial agents. Tobacco, the second leading actual cause of death in the United States, received <10% of the total budget allotted for sexual behavior research. Despite the fact that firearms were responsible for ~40,000 deaths in 2017, firearms research received only $900,000 on average over the last 6 years. In fact, from 2010 to 2014, there was no grant money awarded for firearms research using our search criteria.

**Figure 6:**
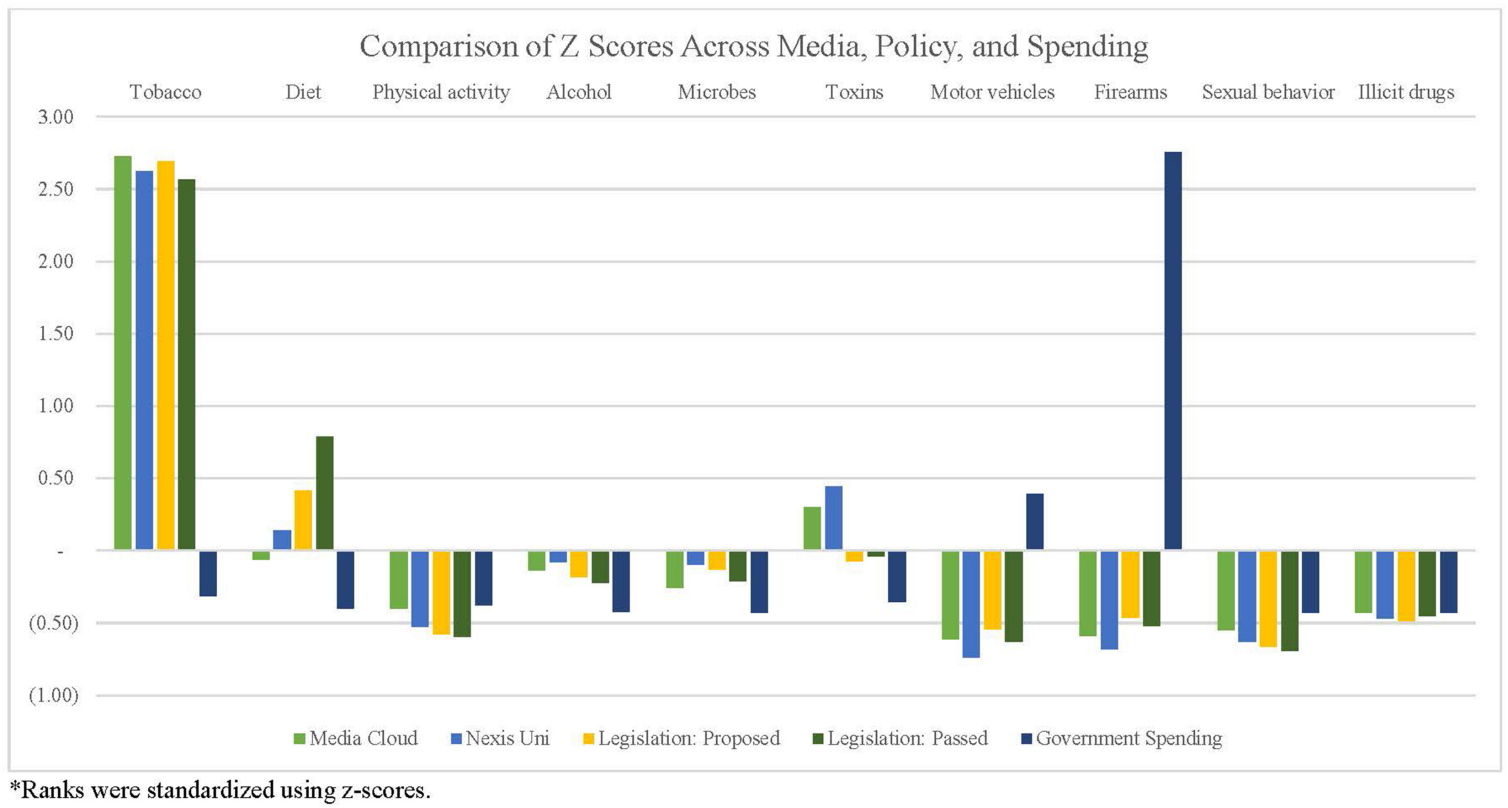
Comparing standardized rank of actual causes of death, media, policy, and funding.

Finally, we compared the rankings and relative weights of the actual causes of death, media attention, policy attention, and government spending over time. Table 2 presents the simple rankings for each cause of death across categories, while Figure 6 presents the findings of standardized rankings, which were calculated using z-scores. This allows for a direct comparison of each cause of death relative to the amount of attention received in media, policy, and government funding. For example, the ratio between tobacco-related deaths (440,000 deaths/year) and tobacco-related Media Cloud attention (168,706 occurrences/year) resulted in a relatively high, positive z-score. The ratio of deaths and Media Cloud attention related to poor diet (503,000 deaths/year and 1,103,447 occurrences/year), on the other hand, resulted in a slight, negative z-score. This figure highlights the magnitude of misalignment between media, policies, and government spending, with larger deviations—either positive or negative—indicating more substantial misalignments.

**Table 2:** Comparing rank of actual causes of death, media, policy, and funding.

**Rank**	**Actual cause of death**	**Media presence: Media Cloud**	**Media presence: Nexis Uni**	**Policy: #bills proposed**	**Policy: #bills passed**	**Government funding**
1	Poor diet	Poor diet	Poor diet	Poor diet	Physical inactivity	Sexual behavior
2	Tobacco	Motor vehicles	Motor vehicles	Physical inactivity	Poor diet	Illicit drug use
3	Toxic agents	Firearms	Illicit drug use	Illicit drug use	Illicit drug use	Microbial agents
4	Microbial agents	Illicit drug use	Firearms	Toxic agents	Toxic agents	Poor diet
5	Illicit drug use	Physical inactivity	Physical inactivity	Microbial agents	Microbial agents	Alcohol
6	Alcohol	Microbial agents	Toxic agents	Sexual behavior	Motor vehicles	Tobacco
7	Physical inactivity	Toxic agents	Microbial agents	Motor vehicles	Sexual behavior	Toxic agents
8	Firearms	Alcohol	Tobacco	Alcohol	Firearms	Physical inactivity
9	Motor vehicles	Sexual behavior	Alcohol	Firearms	Alcohol	Motor vehicles
10	Sexual behavior	Tobacco	Sexual behavior	Tobacco	Tobacco	Firearms

## Discussion

Examining the totals for the actual causes of death, as well as the representation in media, legislation, and government funding, helps elucidate public health attention and priorities in the United States. Overall, our study suggests that the nation's priorities (as measured by media attention, policy attention, and funding) are not consistently aligned with the actual causes of death. Some, such as sexual behavior, receive relatively little media attention, while others, including alcohol, are given little policy consideration. Finally, some causes of death, particularly firearms, are impacted by larger-scale factors. For example, the political ideology and subsequent restrictions surrounding firearms (e.g., the Dickey Amendment) have likely impacted funding streams (38, 39).

Perhaps the largest misalignment is related to tobacco. Though it was ranked as the second largest actual cause of death in the United States, tobacco consistently ranked lower than many other causes of death in media attention, policy legislation, and allocated research funding. This may be due in part to the longstanding knowledge of tobacco's negative health effects, compared to emerging public health issues, such as illicit drug use (in particular opioid use). The consistently low tobacco ranking may also be a result of the strong political influence of the tobacco industry (40). Finally, the limited presence of tobacco-related focusing events, drivers of policy change that encourage political focus on a particular issue (41, 42), may help to explain the relatively limited emphasis in media, policy, and government spending. Examples of tobacco-related focusing events include the Master Settlement Agreement in 1998, which limited tobacco advertising (43), as well as recent hospitalizations and deaths due to e-cigarette or vaping-associated lung injury (44, 45). Without the presence of such events, chronic diseases may be less likely to find “windows of opportunity” (42) and, consequently, receive media, policy, or funding attention. Overall, these rankings suggest that the resources for some diseases or conditions may be out of proportion with the overall burden (i.e., morbidity and mortality).

Additionally, the Spearman correlations revealed misalignments between the actual causes of death and media, policy, and federal funding. Based on the correlations, legislation may be the most closely aligned with the actual causes of death [proposed: (*r*_s_ = 0.27); passed: (*r*_s_ = 0.19)]. However, the correlations between legislation and the actual causes of death are still only small-to-moderate. It is also worth noting that the *r*-value decreases slightly between proposed legislation and passed legislation. These findings may suggest that lawmakers are attempting to address the actual causes of death but may be encountering difficulties passing legislation. The comparison between Media Cloud and the actual causes of death, on the other hand, revealed a small negative correlation (*r*_s_ = −0.02), suggesting that this outlet may be the least aligned with the actual causes of death.

### Limitation

There are several limitations of our study. First, we acknowledge that mortality is not the only outcome that public health interventions seek to impact but rather is one easily accessible in studies like this one. Though this study selected mortality as its outcome of interest, other factors (e.g., morbidity and socio-political context) also influence the prioritization of public health resources. Another possible limitation of this study relates to the selected databases. For example, while both Media Cloud and Nexis Uni were chosen to provide an extensive, comprehensive approach to media, these databases do not capture all forms of media attention. As a result, there were likely pertinent data not captured in this analysis, which may have impacted the results. Data identified in this study—in media, policy, and government spending—were analyzed based on quantity, and each occurrence was given an equal weight. Future studies may explore the reach or quality of data from these databases (e.g., placing a relative weight on individual social media shares compared to a national news outlet). It is also important to acknowledge that this paper focused only on the proposal and implementation of federal-level policies. Though state and local-level policies can significantly impact public health outcomes (46), we believe that shifting the nation's focus as a whole will need to include a significant focus at the federal level. Future studies may explore and contrast state and local-level policy and media attention in relation to the actual causes of death. In addition, as noted in previous studies (47), due to the complexity and variation across agencies, as well as difficulty accessing data, we were unable to find public health spending costs for specific causes of death. As a result, we chose to use the TAGGS database as a proxy for national-level public health spending. Additionally, though we suggest multipronged approaches to address the actual causes of death, we recognize that increased media attention, national policies, and government spending are not causally linked with lower levels of actual causes of deaths. However, as we have described, similar approaches have demonstrated promising results, including decreased morbidity and mortality (29). Lastly, a limitation of this paper was that it did not account for lobbying funds related to specific causes of death. Research has shown that lobbying can impact legislators' voting and, consequently, available funding (46, 48–51). However, analyzing lobbying funds was beyond the scope of this project but could be addressed in future work. Despite these limitations, this study adds to the existing policy literature by exploring media coverage, policy attention, and government spending as indicators of public health attention and priorities in the United States.

Based on the results of their study, McGinnis and Foege called for a greater investment in prevention, thus shifting away from a disease treatment approach (30). Likewise, Mokdad et al. described the substantial number of deaths attributable to poor diet, physical inactivity, and tobacco and suggested that a more preventative approach to health may impact mortality (31). In a related study, Vargas and colleagues found that the leading causes of death in the United States are under-represented in the NIH prevention research portfolio (52). This study revealed similar findings regarding the actual causes of death in the United States. Additionally, it highlighted incongruences between the actual causes of death and media attention, policy attention, and government spending.

There are two primary implications that arise from the findings of this study. First, there is a need be sure that actual causes of death are receiving adequate attention. As previous researchers have noted, prioritizing the use of limited resources is imperative for public health impact (53). Unfortunately, resources in the United States—media, policy, and funding channels—are currently prioritizing different issues, leading to incongruences in the nation's focus on public health. The misalignments across media, policy, and funding sectors may be related to stakeholders' vested interests (e.g., generating interest in news stories vs. satisfying constituents).

When we consider redistributing limited resources, it is important to clarify that we are not simply advocating for the shifting of resources from one preventable cause of death in favor of another. Public health is often a zero-sum game (i.e., the total amount for prevention is relatively fixed over time), and decreasing mortality rates is far more complex than simply redistributing resources. Instead, we are acknowledging that the actual causes of death would be best addressed through prevention (54), and, unfortunately, resources dedicated to prevention constitute only a small portion of total healthcare spending. In 2017, roughly 95% of healthcare spending was treatment-focused, leaving only 5% for prevention and public health efforts (55). The U.S. healthcare system is primarily focused on medical treatment; however, a shift toward prevention would result in a more effective and efficient use of resources (47). This is particularly challenging in public health, due to the reliance on resource-based planning, which often prioritizes immediate health needs (i.e., medical treatment) over long-term challenges (56). The results of disease prevention efforts may not be immediately apparent, thus diminishing the perceived sense of urgency by stakeholders and the public. Given the limited resources designated for public health efforts, it may be beneficial to maximize the impact of resources to better align with the actual causes of death in the United States. As discussed previously, this approach has demonstrated success in the past (29).

The second implication of this work is the potential benefit of intervening across multiple areas—through media attention, national legislation, and government spending—to impact mortality rates in public health. As we have seen from our approach, coverage in the media does not always correlate with policy and funding action. For example, deaths related to firearms receive a moderate amount of media attention; however, without national-level policy changes and funding increases, the number of annual deaths is unlikely to change. As previous research has suggested, to affect change, we need to intervene at multiple loci of control, involving stakeholders from multiple public health sectors (57). If, for example, media attention, federal policy, and government spending each better addressed tobacco-related deaths—increasing awareness through the media, proposing and passing federal-level legislation, and increasing research surrounding tobacco prevention and cessation—there would be the potential to shift priorities and better address preventable deaths.

Collaboration between researchers and policymakers could be helpful when developing priorities. For example, researchers could strive to more clearly define public health problems and engage in early stages of the policy process; they could also more effectively communicate research findings with both media representatives and policymakers to improve evidence-based policymaking (58–60). We recognize that public health and policy researchers are acting within a broader political, social, and economic context, but these action steps may help address the misalignments between media attention, policy attention, government spending, and the leading actual causes of death. Such coordinated and comprehensive efforts on a national scale could be used to improve health and decrease death totals for many—if not all—of the actual causes of death. However, mobilizing support in these different arenas requires a significant investment, as multilevel priority changes are often difficult undertakings (57). Additionally, we recognize that decentralized government and industry influences on policy in the United States pose a significant barrier to these efforts.

Ultimately, prevention efforts are not a high enough priority in the United States. A national shift toward population-based planning, wherein long-term health outcomes were considered before allocating resources, would encourage a more prevention-centered health system (47). Until then, however, those invested in the nation's health must make the most of the available resources and prioritize public health through media, policy, and government funding.

## Data Availability Statement

The original contributions presented in the study are publicly available. This data can be found here: 1. Institute for Health Metrics and Evaluation—Available from: http://www.healthdata.org/ 2. Media Cloud—Available from: https://mediacloud.org 3. Nexis Uni—Available from: https://www.lexisnexis.com 4. Congress.gov—Available from: https://www.congress.gov 5. Tracking Accountability in Government Grants Systems—Available from: https://taggs.hhs.gov/.

## Author Contributions

MP: formal analysis and writing-original draft preparation. AE and SM-R: writing-review and editing. RB: conceptualization, supervision, and writing-review and editing. All authors contributed to the article and approved the submitted version.

## Conflict of Interest

The authors declare that the research was conducted in the absence of any commercial or financial relationships that could be construed as a potential conflict of interest.
