# Supplementary material for: Actual Causes of Death in Relation to Media, Policy, and Funding Attention: Examining Public Health Priorities
Source: Front Public Health. 2020 Jul 7;8:279. doi: 10.3389/fpubh.2020.00279 (PMC7358349; doi:10.3389/fpubh.2020.00279)
Supplement: Supplementary file 1 [file Table_1.DOCX]

**Supplementary Table 1:** Search Terms for Each Cause of Death

| **Cause of Death** | **Search Terms** |
| --- | --- |
| Diet | diet* OR food* OR "healthy food" OR "healthy eating" OR nutrition OR “fruits and vegetables” OR fruit* OR vegetable* OR nutrient* OR nutrition OR fat OR calorie OR caloric OR overweight OR obese OR BMI |
| Tobacco | tobacco OR smoking OR cigarette* OR vaping OR nicotine OR cigar* OR “secondhand smoke” OR “secondhand smoking” |
| Toxins | toxin* OR toxic OR exposure OR pollut* OR asbestos OR “lead poisoning” OR benzene OR “air pollution” OR ozone OR “carbon monoxide” OR particulat* |
| Microbes | microb* OR bacteri* OR fung* OR algae OR protozoa OR virus* OR viral OR parasite* OR vaccinat* OR immuniz* OR infect* OR flu OR influenza OR pneumonia OR tuberculosis |
| Illicit drugs | “illicit drugs” OR drug* OR “street drug” OR “street drugs” OR “controlled substance” OR “controlled substances” OR “drug abuse” OR “recreational drugs” OR “substance abuse” |
| Alcohol | alcohol OR alcoholism OR alcoholic OR “alcohol abuse” OR “binge drinking” OR cirrhosis |
| Physical activity | exercis* OR fitness OR “physical activity” OR “physical inactivity” OR “physical fitness” OR workout OR overweight OR obese OR BMI |
| Firearms | firearm* OR gun* OR handgun* OR semiautomatic OR pistol* OR rifle* OR shotgun* OR ammunition* OR ballistics OR gunshot* |
| Motor vehicles | (vehicle* OR car* OR automobile* OR truck* OR van*) AND (accident OR crash OR injur* OR “seat belt” OR “safety seat”) |
| Sexual behavior | (sex* OR “sexual behavior” OR “sex behavior” OR “sexual activity” OR “oral sex” OR “anal sex” OR “men who have sex with men” OR “unprotected sex”) AND (health* OR disease* OR infection* OR STD OR STI OR HIV OR AIDS OR hepatitis OR cancer* OR safe*) |
