# Supplementary material for: Actual Causes of Death in Relation to Media, Policy, and Funding Attention: Examining Public Health Priorities
Source: Front Public Health. 2020 Jul 7;8:279. doi: 10.3389/fpubh.2020.00279 (PMC7358349; doi:10.3389/fpubh.2020.00279)
Supplement: Supplementary file 2 [file Table_2.DOCX]

**Supplementary Table 2:** Database Search Strategies

| **Database** | **Notes** |
| --- | --- |
| Institute for Health Metrics and Evaluation (IHME) | Website: <https://vizhub.healthdata.org/gbd-compare/>  Select the following using advanced settings:   - Single - Display = Risk/Cause - Rank = Cause - Level = 2 - Measure = Deaths - Location = United States - Year = 2017 - Age = All ages - Sex = Both - Unites = # - Value = Observed   The actual causes of death were measured using the following IHME categories:   - Tobacco = Risk = Behavioral = “tobacco” = 440,359 - Diet = Risk = Behavioral = “dietary risks” = 503,390 - Physical activity = Risk = Behavioral = “low physical activity” = 72,957 - Alcohol = Risk = Behavioral = “alcohol use” = 80,985 - Microbes = Cause = Communicable, maternal, neonatal, and nutritional diseases = “respiratory infections and TB” and “enteric infections” and “other infectious” and “NTDs and malaria” = 112,513 - Toxins = Risk = Environmental/occupational = “particulate matter pollution” and “occupational carcinogens” and “lead exposure” and “ambient ozone pollution” and “occupational particulate matter, gases, and fumes” and “residential radon” and “other environmental risks” = 201,229 - Motor vehicles = Cause = Injuries = “motor vehicle road injury” = 30,230 - Firearms = Cause = Injuries = “unintentional firearm” and “self-harm” and “physical violence” = 40,229 - Sexual behavior = Risk = Behavioral = “unsafe sex” = 14,388 - Illicit drugs = Risk = Behavioral = “drug use” = 104,937 |
| MediaCloud | Website: <https://mediacloud.org/>  Enter search terms [see S1 Table]  Narrow search results by the following settings:   - Restrict my geographic media sources = United States national and state/local sources - Restrict by time frame |
| NexisUni | Website: [https://advance.lexis.com](https://advance.lexis.com/)  Enter search terms [see S1 Table]  Narrow search results by the following settings:   - Content collection = News - Language = English - Location by publication = “United States” and 50 U.S. states and “District of Columbia” - Publication type = “Newspapers” and “Newswires and press releases” - Geography by document: North American 🡪 United States - Restrict by time frame |
| Congress.gov | Website: <https://www.congress.gov/>  Enter search terms [see S1 Table]  Narrow search results by the following settings:   - Limit search to: Legislation   Status of legislation = “Introduced” OR “Became law”   - Restrict by timeframe |
| TAGGS | Website: <https://taggs.hhs.gov/SearchAward>  Note: Truncating and Boolean phrases are not permitted in this database, so modified search terms were created.  Search terms:   - Tobacco = tobacco smoking cigarette vaping nicotine cigar - Diet = diet eating nutrition fruit vegetable nutrient fat calorie caloric overweight obese BMI - Physical activity = exercise fitness “physical activity” workout overweight obese BMI - Alcohol = alcohol alcoholism alcoholic “binge drinking” cirrhosis - Microbes = microbe microbial bacteria bacterial fungi fungus fungal algae protozoa viral parasite parasitic vaccinate vaccination vaccine immunize immunization infect infection infectious flu influenza pneumonia tuberculosis - Toxins = toxin toxic exposure pollute pollution asbestos “lead poisoning” benzene “air pollution” ozone “carbon monoxide” particulate - Motor vehicles = vehicle automobile truck - Firearms = handgun firearm semiautomatic pistol rifle ammunition ballistics gunshot rifle - Sexual behaviors = sex sexual sexually anal STD STI HIV AIDS HPV hepatitis condom - Illicit drugs = substance opioid drug-free   Narrow search results by the following settings:   - Issue Date FY - 50 U.S. states and “District of Columbia” - Restrict by timeframe |
