# Supplementary material for: Actual Causes of Death in Relation to Media, Policy, and Funding Attention: Examining Public Health Priorities
Source: Front Public Health. 2020 Jul 7;8:279. doi: 10.3389/fpubh.2020.00279 (PMC7358349; doi:10.3389/fpubh.2020.00279)
Supplement: Supplementary file 3 [file Table_3.DOCX]

**Supplementary Table 3:** Media Presence for Individual Causes of Death in Media Cloud 2010-2019

| **Cause of Death** | **2010** | **2011** | **2012** | **2013** | **2014** | **2015** | **2016** | **2017** | **2018** | **2019** | **Yearly Average** |
| --- | --- | --- | --- | --- | --- | --- | --- | --- | --- | --- | --- |
| Poor diet | 114,803 | 322,288 | 348,604 | 569,623 | 683,419 | 763,462 | 918,895 | 1,363,371 | 2,986,018 | 2,963,984 | 1,103,447 |
| Tobacco | 20,734 | 53,760 | 58,555 | 92,945 | 120,765 | 124,100 | 146,275 | 189,850 | 406,201 | 473,877 | 168,706 |
| Toxic agents | 32,813 | 80,583 | 80,708 | 133,973 | 169,304 | 196,586 | 253,232 | 343,286 | 724,894 | 729,990 | 274,537 |
| Microbial agents | 32,670 | 87,885 | 104,777 | 166,490 | 265,822 | 263,458 | 371,560 | 435,116 | 991,343 | 981,953 | 370,107 |
| Illicit drug use | 67,606 | 174,416 | 190,767 | 304,895 | 372,015 | 430,961 | 562,894 | 775,261 | 1,689,885 | 1,618,172 | 618,687 |
| Alcohol | 21,645 | 58,137 | 67,082 | 108,342 | 126,142 | 149,147 | 175,340 | 257,995 | 557,827 | 506,654 | 202,831 |
| Physical inactivity | 39,706 | 103,731 | 117,013 | 187,802 | 238,464 | 279,100 | 361,562 | 494,899 | 1,051,722 | 933,297 | 380,730 |
| Firearms | 79,769 | 223,880 | 265,025 | 456,245 | 480,124 | 624,121 | 833,699 | 984,870 | 2,366,735 | 2,097,891 | 841,236 |
| Motor vehicles | 100,910 | 280,880 | 302,058 | 506,050 | 601,079 | 698,723 | 877,763 | 1,314,994 | 2,528,153 | 2,606,324 | 981,693 |
| Sexual behavior | 18,118 | 44,537 | 57,747 | 87,048 | 105,894 | 125,779 | 179,833 | 245,520 | 539,239 | 481,836 | 188,555 |
