# Supplementary material for: Actual Causes of Death in Relation to Media, Policy, and Funding Attention: Examining Public Health Priorities
Source: Front Public Health. 2020 Jul 7;8:279. doi: 10.3389/fpubh.2020.00279 (PMC7358349; doi:10.3389/fpubh.2020.00279)
Supplement: Supplementary file 4 [file Table_4.DOCX]

**Supplementary Table 4:** Media Presence for Individual Causes of Death in Nexis Uni 2010-2019

| **Cause of Death** | **2010** | **2011** | **2012** | **2013** | **2014** | **2015** | **2016** | **2017** | **2018** | **2019** | **Yearly Average** |
| --- | --- | --- | --- | --- | --- | --- | --- | --- | --- | --- | --- |
| Poor diet | 301,796 | 326,417 | 323,094 | 327,653 | 325,525 | 316,202 | 286,982 | 282,441 | 286,962 | 53,457 | 283,053 |
| Tobacco | 72,608 | 78,863 | 80,332 | 80,946 | 79,274 | 78,878 | 69,271 | 68,703 | 71,550 | 9,847 | 69,027 |
| Toxic agents | 96,570 | 100,903 | 92,408 | 93,556 | 98,078 | 94,729 | 89,527 | 87,545 | 91,682 | 17,065 | 86,206 |
| Microbial agents | 80,621 | 86,498 | 89,878 | 89,090 | 113,268 | 102,512 | 93,552 | 80,976 | 86,407 | 16,255 | 83,906 |
| Illicit drug use | 163,413 | 172,205 | 182,039 | 180,325 | 173,660 | 176,209 | 173,497 | 176,598 | 191,091 | 29,063 | 161,810 |
| Alcohol | 65,230 | 68,278 | 70,287 | 71,193 | 65,809 | 66,197 | 58,669 | 57,698 | 60,217 | 6,756 | 59,033 |
| Physical inactivity | 151,307 | 165,556 | 165,016 | 162,467 | 153,957 | 148,928 | 133,475 | 128,505 | 127,481 | 22,322 | 135,901 |
| Firearms | 159,093 | 174,104 | 183,828 | 206,767 | 172,039 | 182,791 | 179,365 | 154,407 | 173,092 | 17,835 | 160,332 |
| Motor vehicles | 218,689 | 242,429 | 243,215 | 247,158 | 233,203 | 236,512 | 212,499 | 199,161 | 182,266 | 41,430 | 205,656 |
| Sexual behavior | 36,378 | 38,708 | 44,028 | 45,907 | 46,978 | 47,780 | 49,108 | 47,012 | 53,814 | 5,694 | 41,541 |
