# Supplementary material for: Actual Causes of Death in Relation to Media, Policy, and Funding Attention: Examining Public Health Priorities
Source: Front Public Health. 2020 Jul 7;8:279. doi: 10.3389/fpubh.2020.00279 (PMC7358349; doi:10.3389/fpubh.2020.00279)
Supplement: Supplementary file 5 [file Table_5.DOCX]

**Supplementary Table 5:** Proposed Policy Totals for Individual Causes of Death 2010-2019

| **Cause of Death** | **2009-2010** | **2011-2012** | **2013-2014** | **2015-2016** | **2017-2018** | **2019-2020** | **Yearly Average** |
| --- | --- | --- | --- | --- | --- | --- | --- |
| Poor diet | 1,487 | 1,133 | 1,180 | 1,258 | 1,552 | 1,435 | 1,341 |
| Tobacco | 402 | 317 | 292 | 437 | 546 | 391 | 398 |
| Toxic agents | 1,098 | 891 | 760 | 884 | 967 | 919 | 920 |
| Microbial agents | 667 | 507 | 459 | 584 | 378 | 756 | 559 |
| Illicit drugs | 1,211 | 974 | 937 | 1,248 | 1543 | 1,366 | 1,213 |
| Alcohol | 414 | 365 | 330 | 489 | 592 | 448 | 440 |
| Physical inactivity | 1,434 | 1,153 | 1,216 | 1,321 | 1,414 | 1,064 | 1,267 |
| Firearms | 319 | 298 | 375 | 520 | 608 | 447 | 428 |
| Motor vehicles | 324 | 225 | 198 | 262 | 866 | 797 | 445 |
| Sexual behavior | 404 | 342 | 405 | 458 | 702 | 696 | 501 |
