# Supplementary material for: Actual Causes of Death in Relation to Media, Policy, and Funding Attention: Examining Public Health Priorities
Source: Front Public Health. 2020 Jul 7;8:279. doi: 10.3389/fpubh.2020.00279 (PMC7358349; doi:10.3389/fpubh.2020.00279)
Supplement: Supplementary file 6 [file Table_6.DOCX]

**Supplementary Table 6:** Passed Policy Totals for Individual Causes of Death 2010-2019

| **Cause of Death** | **2009-2010** | **2011-2012** | **2013-2014** | **2015-2016** | **2017-2018** | **2019-2020** | **Yearly Average** |
| --- | --- | --- | --- | --- | --- | --- | --- |
| Poor diet | 49 | 34 | 40 | 53 | 51 | 26 | 42 |
| Tobacco | 29 | 20 | 13 | 13 | 21 | 8 | 17 |
| Toxic agents | 51 | 37 | 29 | 34 | 39 | 22 | 35 |
| Microbial agents | 41 | 21 | 23 | 33 | 18 | 19 | 26 |
| Illicit drugs | 56 | 43 | 38 | 41 | 51 | 20 | 42 |
| Alcohol | 32 | 21 | 13 | 14 | 24 | 9 | 19 |
| Physical inactivity | 78 | 47 | 40 | 60 | 56 | 17 | 50 |
| Firearms | 30 | 19 | 15 | 21 | 23 | 11 | 20 |
| Motor vehicles | 35 | 18 | 11 | 20 | 50 | 18 | 25 |
| Sexual behavior | 24 | 12 | 15 | 18 | 40 | 12 | 20 |
