# Supplementary material for: Actual Causes of Death in Relation to Media, Policy, and Funding Attention: Examining Public Health Priorities
Source: Front Public Health. 2020 Jul 7;8:279. doi: 10.3389/fpubh.2020.00279 (PMC7358349; doi:10.3389/fpubh.2020.00279)
Supplement: Supplementary file 7 [file Table_7.DOCX]

**Supplementary Table 7:** Federal Funding Totals for Individual Causes of Death 2010-2019*

| **Cause of Death** | **2010** | **2011** | **2012** | **2013** | **2014** | **2015** | **2016** | **2017** | **2018** | **2019** | **Yearly Average** |
| --- | --- | --- | --- | --- | --- | --- | --- | --- | --- | --- | --- |
| Poor diet | $1,566.20 | $1,533.72 | $1,525.71 | $1,420.88 | $933.87 | $923.69 | $954.22 | $933.91 | $1,042.78 | $1,062.36 | $1,189.73 |
| Tobacco | $336.33 | $240.03 | $240.16 | $287.50 | $296.97 | $318.35 | $315.76 | $296.61 | $290.85 | $292.10 | $291.46 |
| Toxic agents | $184.83 | $163.22 | $171.41 | $165.52 | $177.71 | $222.50 | $228.83 | $227.08 | $243.69 | $278.95 | $206.37 |
| Microbial agents | $4,541.63 | $3,617.56 | $3,685.62 | $3,341.21 | $3,778.24 | $4,050.20 | $4,336.98 | $4,531.55 | $4,557.63 | $4,778.12 | $4,121.87 |
| Illicit drug use | $3,693.15 | $3,692.67 | $3,775.79 | $3,558.48 | $3,885.07 | $3,928.96 | $4,087.25 | $4,611.31 | $6,015.83 | $6,861.52 | $4,411.00 |
| Alcohol | $764.48 | $727.89 | $842.57 | $656.12 | $630.28 | $642.67 | $656.30 | $700.35 | $799.16 | $800.81 | $722.06 |
| Physical inactivity | $100.52 | $88.46 | $91.96 | $92.32 | $89.85 | $92.23 | $92.06 | $102.23 | $114.29 | $134.85 | $99.88 |
| Firearms | $- | $- | $- | $- | $- | $0.43 | $1.52 | $2.15 | $ 3.89 | $1.82 | $ 0.98 |
| Motor vehicles | $3.25 | $ 4.39 | $3.32 | $3.25 | $1.56 | $1.36 | $1.92 | $2.05 | $3.43 | $3.96 | $2.85 |
| Sexual behavior | $5,853.42 | $4,935.47 | $4,825.61 | $4,699.00 | $5,023.42 | $4,948.43 | $6,857.95 | $6,132.02 | $5,911.61 | $5,356.47 | $5,454.34 |

*Amounts listed in millions
